# Model-independent quantum phases classifier

**DOI:** 10.1038/s41598-023-33301-0

**Published:** 2023-09-02

**Authors:** F. Mahlow, F. S. Luiz, A. L. Malvezzi, F. F. Fanchini

**Affiliations:** Faculty of Sciences, UNESP-São Paulo State University, 17033-360 Bauru, SP Brazil

**Keywords:** Phase transitions and critical phenomena, Computational methods

## Abstract

Machine learning has transformed science and technology. In this article, we present a model-independent classifier that uses the *k*-Nearest Neighbors algorithm to classify phases of a model for which it has never been trained. This is done by studying three different spin-1 chains with some common phases: the XXZ chains with uniaxial single-ion-type anisotropy, the bond alternating XXZ chains, and the bilinear biquadratic chain. We show that the algorithm trained with two of these models can, with high probability, determine phases common to the third one. This is the first step towards a universal classifier, where an algorithm can recognize an arbitrary phase without knowing the Hamiltonian, since it knows only partial information about the quantum state.

## Introduction

The interest on quantum phases and phase transitions has been recently renewed due to new physics uncovered by experiments on cuprate superconductors, heavy fermion materials, organic conductors, and other strongly correlated materials^1^. From a theoretical perspective, low-dimensional quantum lattice models can capture many aspects of these new phenomena^2,3^. Therefore, determining the quantum (i.e., groundstate) phase diagrams of these models is an important challenge in condensed matter and statistical physics^4^.

Concerning quantum phases classification, many alternatives have been highlighted as promising^5–9^ with special emphasis on machine learning (ML). The main difference between ML and other statistical models is the fact that an algorithm can improve its performance, that is, learn, without the need for such explicit programming^10,11^. ML is a form of applied statistics, where computers use data (usually in large quantities) to estimate functions with a high degree of complexity, which can be used to make predictions and observe patterns in these data sets^12,13^. ML has been widely used in the physical sciences, including cosmology^14–16^, quantum information^17–19^, many-body physics^8^, and also to classify quantum phases and detect their transitions^20–27^.

In this manuscript, we analyze the correlation between spins in a closed chain for three distinct spin-1 models. We show that these correlations hold information about the phases of these distinct models, and there is considerable overlap between phase information of different models. This explains how ML algorithms, training a known model, are capable of detecting some phases of another unknown model. To illustrate this, we use a machine learning classifier, fed with correlation and phase labels of two known models, to detect the phases of a third unknown model. We show that the prediction succeeds when the overlap of the information about the different phases is minimal. Also, we show that it is possible to apply a transformation in the dataset of the correlations, which allows minimizing the overlap of the information about the different phases, making the ML predictor more accurate.

## Physical models

To develop our studies, we use three well-known distinct spin-1 models, where the phase diagram of these models and their central features are well established in the literature^28–30^. The first model, we present is the *(1) XXZ chains with uniaxial single-ion-type anisotropy*, whose Hamiltonian is given by:

1 $$\begin{aligned} \mathcal{H}_{1} = \sum _{l=1}^{N} \left[ J \left( {S_l^x}{S_{l+1}^x}+{S_l^y}{S_{l+1}^y}\right) +J_z{S_l^z}{S_{l+1}^z}\right] + D\sum _{l=1}^{N} {S_l^z}^2, \end{aligned}$$

where $S_l$ is a spin-1 operator acting on site *l* of a one-dimensional lattice (chain) with *N* sites, *D* represents uniaxial single ion anisotropy, and $J(=1), J_z$ are spin couplings. For Hamiltonian Eq. (1), the phase diagram consists of six distinct phases, namely, Haldane, Large D, XY1, XY2, Ferromagnetic, and a Néel, and several different transitions can occur^28^. The next model is the *(2) bond-alternating XXZ chain*, whose Hamiltonian is given by:

2 $$\begin{aligned} \mathcal{H}_2 = \sum _{l=1}^{N} \left[ 1-\delta (-1)^l \right] \left[ {S_l^x}{S_{l+1}^x} + {S_l^y}{S_{l+1}^y} + \Delta {S_l^z}{S_{l+1}^z}\right] , \end{aligned}$$

where $\Delta$ is the strength of the Ising-type anisotropy that originates from the spin-orbit interaction in magnetic materials and $\delta$ is the alternation of the bond that describes dimerization. The phase diagram of the model Eq. (2) shows the Ferromagnetic phase, XY1, Néel, Haldane, and Dimerized^29^. Finally, the last model analyzed was the *(3) bilinear biquadratic chain*, whose Hamiltonian is given by:

3 $$\begin{aligned} \mathcal{H}_3 = \sum _{l=1}^{N} \left[ \cos \theta (S_l.S_{l+1})+ \sin \theta (S_l.S_{l+1})^2\right] , \end{aligned}$$

where $\theta \in [0, 2 \pi )$ quantifies the amount of coupling between the nearest neighboring spins. The model Eq. (3) presents four phases, namely, Haldane, Trimerized, Ferromagnetic, and Dimerized^30^.

To illustrate and summarize the phases and the common phases contained among these models, we prepare Table 1. As we can note, all phases of $\mathcal{H}_2$ are contained in the combined phases of $\mathcal{H}_1$ and $\mathcal{H}_3$, being the only phase diagram in which this occurs. The union of $\mathcal{H}_1$ and $\mathcal{H}_2$ contains three of four phases of $\mathcal{H}_3$, and the union of $\mathcal{H}_2$ and $\mathcal{H}_3$ contains four of six phases of $\mathcal{H}_1$. In total, we have five phases that are shared by at least two models (Haldane, Néel, Ferromagnetic, XY1, and Dimer) and three phases unique to a single model (Large-D, XY2, and Trimer).

We assume that even when different models appear on the phase diagram, a given phase has a trademark that is model independent. Here, we propose that the spin correlations can capture such a signature. To test this hypothesis, we will analyze several correlations between the spins in the chain.

**Table 1:**
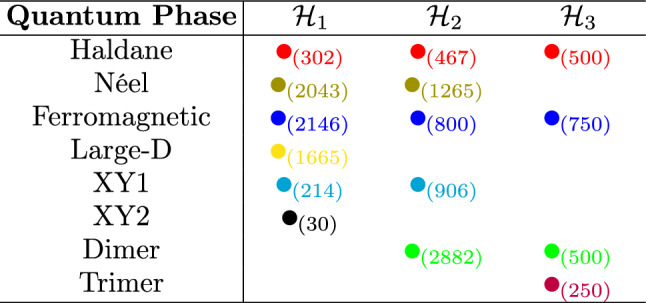
Phases contained in the diagrams corresponding to the three Hamiltonians analyzed.

The filled circles indicate that the quantum phase is present in the model. The numbers subscribed in the circles represent the amount of data calculated for each phase of each model.

## Data structure

The correlations between neighbors in the closed spin chain are given by the expected values of the following observables: $$\langle S_{1}^{k} S_{i}^{k}\rangle $$ and $$\left\langle \prod _{j} S_{j}^{k}\right\rangle $$, with $$k=\{ x,y,z\}$$, $$ i = [1, N/2+1]$$, $$j=[1,N]$$, and here we consider N = 12 sites. Furthermore, $$\left\langle S_{1}^{k} S_{i}^{k}\right\rangle = \langle \lambda _{0}| S_{1}^{k} S_{i}^{k}|\lambda _{0}\rangle $$, are the expected values of the correlation for the lowest energy state of the Hamiltonian^25^, where $$|\lambda _0\rangle $$ is the ground state. Notice that since the chain is closed and, consequently, the chain properties are cyclic, any non-redundant correlation between two spins is obtained for $$\left\langle S_{1}^{k} S_{i}^{k}\right\rangle $$ with $$ i = [1, N/2+1]$$. The choice of these features is justified because, while two-point correlators identify simple local orders, string correlators captures non-local orders, which is important for the generalization of the method.

To generate the correlation dataset, we considered thousands of different values for the parameters of the Hamiltonians $\mathcal{H}_1$, $\mathcal{H}_2$, and $\mathcal{H}_3$. For $\mathcal{H}_1$, Eq. (1), we range the parameters $J_{z}$ and *D* in the interval $[-\,4,4]$ with a step size of 0.1, this generates a dataset with 6400 data points. For the Hamiltonian $\mathcal{H}_2$, Eq. (2), we range the parameters, $\Delta$ in an interval [0, 1], and the parameters $\delta$ in an interval $[-\,1.5,2.5]$, with step sizes of 0.005 and 0.0125, respectively, this generates a dataset with 6320 data points. Finally, for the Hamiltonian $\mathcal{H}_3$, Eq. (3), we set the range of parameter $\theta$ in the interval $[0,2\pi ]$, with the step size of $\pi \times 10^{-3}$, which results in a dataset with 2000 data points. The presented results have been obtained by means of the exact diagonalization of the Hamiltonians. The labels of the phases for the Hamiltonians $\mathcal{H}_1$, $\mathcal{H}_2$, and $\mathcal{H}_3$, are obtained from the literature^28,29^, and^30^, respectively.

**Figure 1:**
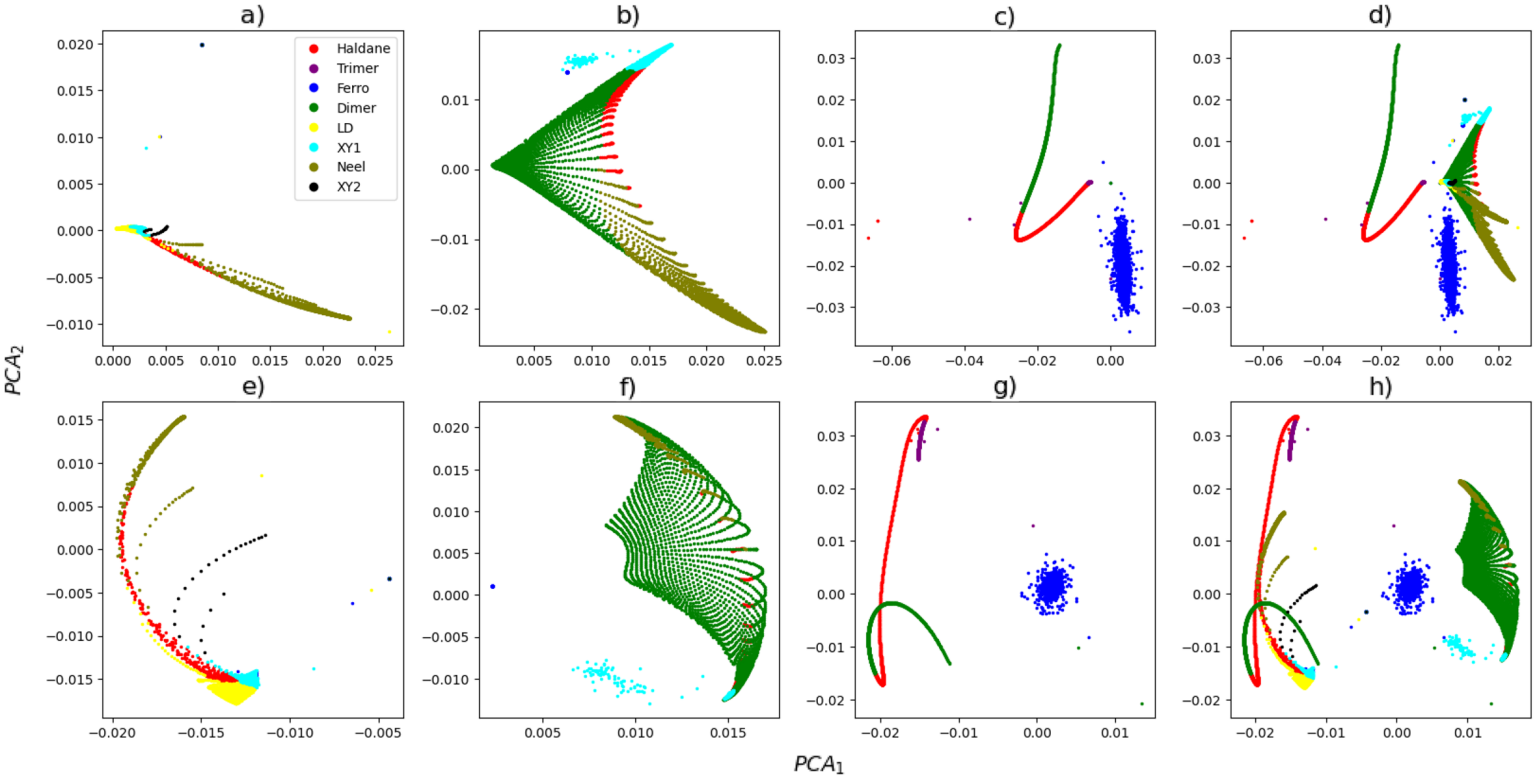
PCA component 1 vs PCA component 2, the color represents the phase of the corresponding data point. And the figures (**a**) and (**e**) refer to the system described by $\mathcal{H}_{1}$, (**b**) and (**f**) refers to $\mathcal{H}_{2}$, (**c**) and (**g**) refers to $\mathcal{H}_{3}$, (**d**) and (**h**) are the data from the three systems. The figures (**a**)–(**d**) are the raw data, and (**e**)–(**h**) are the data after performing the transformation.

With the dataset of the three models, we could visualize the relation of the correlations with the quantum phases. Since we intend to use a classifier algorithm, the idea is to separate, in the multidimensional space, distinct phases in distinct positions. For 12 sites, for example, using the set of correlations described above, we have 24 distinct correlations (a space with 24 dimensions) and to visualize this amount of information in a 3D space is complicated. In order to gain insight into the behavior of the data in the 24-dimensional space, we employed the Principal Component Analysis (PCA) algorithm to reduce the dataset to two dimensions, enabling visualization on a plane. PCA is a multivariate analysis technique that aims to decrease the dimensionality of data while retaining the majority of variation present in the data. The technique utilizes a linear transformation of the original data, resulting in a new set of variables known as principal components^31^. In Fig. 1 we show the two components obtained using the method. With $PCA_1$ on the *X* axis and $PCA_2$ on the *Y*, looking for the best graphical representation of what happens in the correlation space. Note that $PCA_1$ and $PCA_2$ are calculated for all dataset, where we consider different values for the parameters of the Hamiltonians $\mathcal{H}_1$, $\mathcal{H}_2$, and $\mathcal{H}_3$.

One important aspect to consider when working with machine learning is a dataset transformation. In many cases, appropriate transformations can separate the classes in the feature space, which facilitates the classification process. In Fig. 1-a to d we plot the raw data, and in Fig. 1-e to h we plot the raw data after scaling the dataset, for each model, to have a unit norm. This is a well-known renormalization procedure called spatial sign preprocessing^32^. Analyzing Fig. 1, we see that even with the information contained in a single pair of correlations, we are able to separate, with high distinctness, states with different phases. Indeed, there is some overlap of this information for different phases, especially when considering several different models, Fig. 1-d and Fig. 1-h. Moreover, for the normalized data, Fig. 1-e to h, the overlap of information about the phases decreases for all models, almost disappearing for $\mathcal{H}_3$, Fig. 1-g.

Despite the improvement in reducing the overlap, Fig. 1-h shows that there are still overlaps of information from the Haldane phase with Large-D, from the XY1, XY2, Néel, and Haldane phases with the Dimer phase.

Our main goal is to classify the phases of an unknown model, given the distribution of these phases in the correlation space of other known models. For this purpose, we need an algorithm that, given a new quantum state and, consequently, a position in the correlation space, labels it with the corresponding phase based on the information from other known states, located in the vicinity of the new one in the correlation space. As we show below, even a simple algorithm is capable of performing this task with high accuracy.

## *k*-Nearest Neighbors classifier

In this manuscript, we use a supervised algorithm, i.e. where the learning process occurs through labeled data. For this task, one of the simplest machine learning algorithms is the *k*-Nearest Neighbors classifier (*k*-NN)^33^. Despite its simplicity, it presented a good result in our classification problem, which can be explained by the way the *k*-NN works. When a data point of the unknown model is inserted, the algorithm calculates the Euclidean distance (in this work, but the metric can be changed) of this unknown model data point to the *k*-nearest neighbors. The unknown model data point is classified by a plurality vote of its *k* neighbors, with the unknown model data point being assigned to the class most common among them^33–35^. It is important to note that the proximity between the features in the different models observed in Fig. 1, combined with the concept of how the *k*-NN algorithm works, gives a physical meaning of how the algorithm is able to properly classify the data. The phases are classified given the proximity of the expected values of the observables.

With the exception of the number of neighbors *k* used in the *k*-NN algorithm, all parameters were kept as defaults from the Scikit-learn library^34^. In this work, we use $k=50$ and assume that all neighbors have the same voting weight (it could be assumed that the closer, the greater the voting weight). Also, as stated before, it is necessary to use an appropriate transformation that reduces the overlap between the different phases in the correlation space. Once the transformation decreases the overlap between different phases (such as that made in Fig. 1-e to h), it increases the accuracy of the *k*-NN algorithm. The algorithm utilized the 24 described correlations as its training dataset, while their quantum phase served as the target.

**Figure 2:**
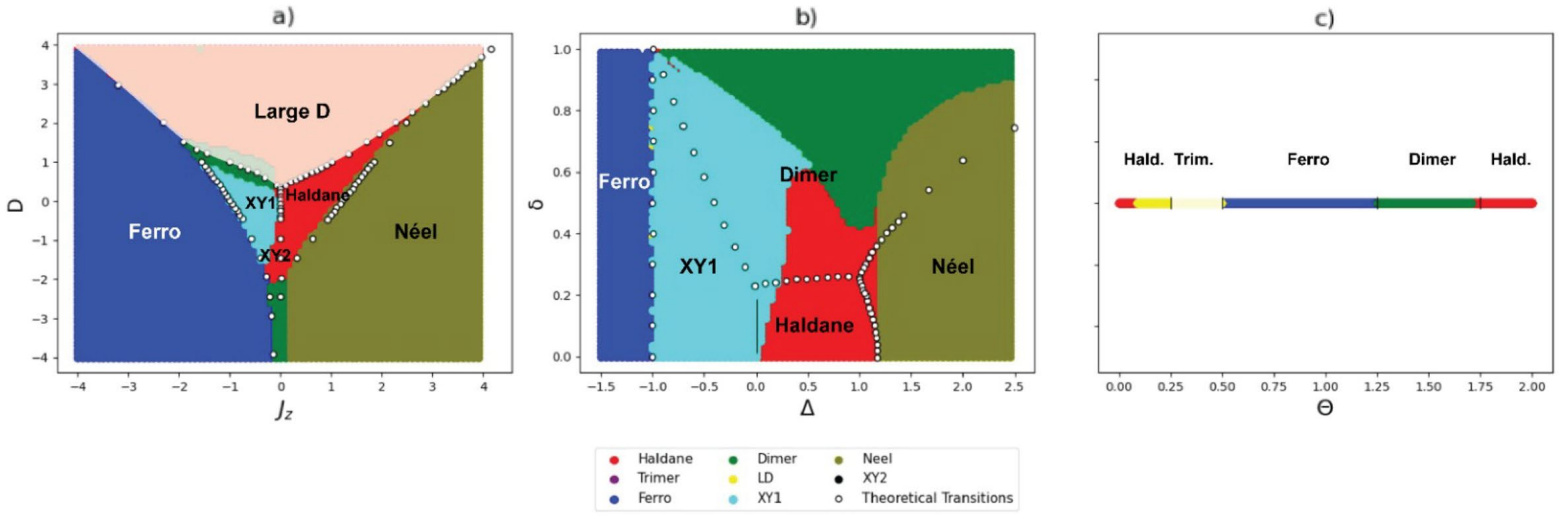
Figure (**a**) shows the phase prediction of model $\mathcal{H}_1$ using the learning of models $\mathcal{H}_2$ and $\mathcal{H}_3$. Figure (**b**) shows the phase prediction of model $\mathcal{H}_2$ using the learning of models $\mathcal{H}_1$ and $\mathcal{H}_3$, and figure (**c**) shows the phase prediction of model $\mathcal{H}_3$ using the learning of models $\mathcal{H}_1$ and $\mathcal{H}_2$. The white dots in panels (**a**) and (**b**), and the vertical lines in panel (**c**) are the theoretical boundaries of phases given in the literature, for models $\mathcal{H}_1$^28^, $\mathcal{H}_2$^29^, and $\mathcal{H}_3$^30^ respectively. The name in black represents the real phase in that place of the model, and the color represents the predicted phase. The phases with transparency (LD in (**a**) and trimer in (**c**)), are the ones that were not present in the algorithm’s training database.

## Results

To test the accuracy of the *k*-NN in transferring learning from the known models to the unknown model, we begin by training the algorithm to classify the data points of $\mathcal{H}_1$. For this case, we train the *k*-NN with data from models $\mathcal{H}_2$ and $\mathcal{H}_3$ (it means that the *k*-NN algorithm knows the dataset of $\mathcal{H}_2$ and $\mathcal{H}_3$ and their respective phase labels), and predict the phases of $\mathcal{H}_1$. In sequence, we do the same for $\mathcal{H}_2$ and $\mathcal{H}_3$ using the data from models $\mathcal{H}_1$ and $\mathcal{H}_3$, and $\mathcal{H}_1$ and $\mathcal{H}_2$, respectively.

It is worth noting, however, that there are phases in $\mathcal{H}_1$ (Large-D and XY2) that do not exist in $\mathcal{H}_2$ and $\mathcal{H}_3$, and a phase in $\mathcal{H}_3$ (Trimer) that does not exist in $\mathcal{H}_1$ and $\mathcal{H}_2$. Clearly, there is no way to learn from the data that were not provided^9,36^, for that reason, a transparency was introduced within the LD phase region to facilitate visual discernment of the algorithm-assigned phase, despite its inherent infeasibility of correct identification. Furthermore, when we predict the phases of $\mathcal{H}_3$, we did the same with the Trimer phase. We do not account for these phases when calculating the accuracy of the algorithm, as they would possibly lead to misleading numbers.

In our analysis, we transformed our dataset following a renormalization procedure called spatial sign preprocessing, where the dataset is scaled to have a unit norm^32^. As stated before, each ground state and its respective labeled phase, are represented by the correlations between a pair of spins, $\left\langle S_{1}^{k},S_{i}^{k}\right\rangle$, which provides $N/2+1$ features for each variable $k=\{x,y,z\}$, and global correlations $\left\langle \prod _{j} S_{j}^{k}\right\rangle$. As we note in Fig. 1, even after the dataset transformation, some overlap between phases is still present. For example, in Fig. 1-f, it is easy to notice the overlap of the XY1 phase with the Haldane and Dimer phases, and the overlap between Néel and Dimer phases. Figure 2 shows the phase prediction of the *k*-NN algorithm considering 12 sites. In Fig. 2-a we estimate the phase diagram of $\mathcal{H}_1$ given the data from $\mathcal{H}_2$ and $\mathcal{H}_3$, in Fig. 2-b we estimate the phase diagram of model $\mathcal{H}_2$ given the data from $\mathcal{H}_1$ and $\mathcal{H}_3$, and analogous in Fig. 2-c where we estimate the phases of the $\mathcal{H}_3$ given the data from $\mathcal{H}_1$ and $\mathcal{H}_2$. The prediction of the phase diagram of $\mathcal{H}_1$, presented in Fig. 2-a, was incredibly successful with an accuracy of 96.77%. All phases are in the correct locations with few mistakes in the phase transitions. As in our two-dimensional illustrative example, the Haldane phase invades the XY1 phase space, and the confusion between the Néel and Dimer phases persists. The region for which the algorithm lacked training data (LD) was almost entirely classified as the Haldane phase. The reason can be seen thanks to the proximity of the phases in Fig. 1-e. and e. When considering $\mathcal{H}_2$, we observe that the algorithm commits mistakes in the separations of the phases, which made its accuracy the lowest, about 73.53%. Despite this, one aspect needs to be emphasized. The training dataset contains information for $\mathcal{H}_1$ and $\mathcal{H}_3$, which includes all 8 distinct phases. Nevertheless, the Large-D, XY2, and Trimer phases were not indicated by the *k*-NN algorithm for all data of $\mathcal{H}_2$, since the algorithm detects all phases correctly, only making mistakes around the boundaries. Finally, the prediction for model $\mathcal{H}_3$ is presented in Fig. 2-c, where we use the $\mathcal{H}_1$ and $\mathcal{H}_2$ models for learning. In this case it incorrectly classifies the Haldane phase, mixing it with the Large-D, a phase that is not even present in the model. Nevertheless, even making this mistake, the algorithm achieves an accuracy of 88.27%. Even though good results are presented, different strategies can be used to increase the prediction accuracy. One is to add new models to the predictor dataset, as adding new information to the *k*-NN would help avoid incorrect phase prediction. The second is to find a transformation that can separate the phase information in the correlation space. Finally, different machine learning algorithms can certainly be implemented to increase the accuracy.

Another component taken into account in our analysis is the influence of the chain size on the accuracy of the algorithm. The results obtained can be observed in Table 2.

**Table 2:** Accuracy obtained by *k*-NN classifying the quantum phases when there is variation in the size of the spin chain.

	$\mathcal{H}_1$ (%)	$\mathcal{H}_2$ (%)	$\mathcal{H}_3$ (%)
4 Spins	97.42	68.14	72.13
8 Spins	97.77	69.14	79.44
12 Spins	96.77	73.53	88.27

As expected, in general the accuracy of the algorithm increased as a function of the number of spins, as it is a better representation of the system from a physical perspective. The only exception to this behavior was with XXZ for 12 Spins, however, all algorithm errors were concentrated in the phase transition region, which naturally is a more complex task for it.

**Figure 3:**
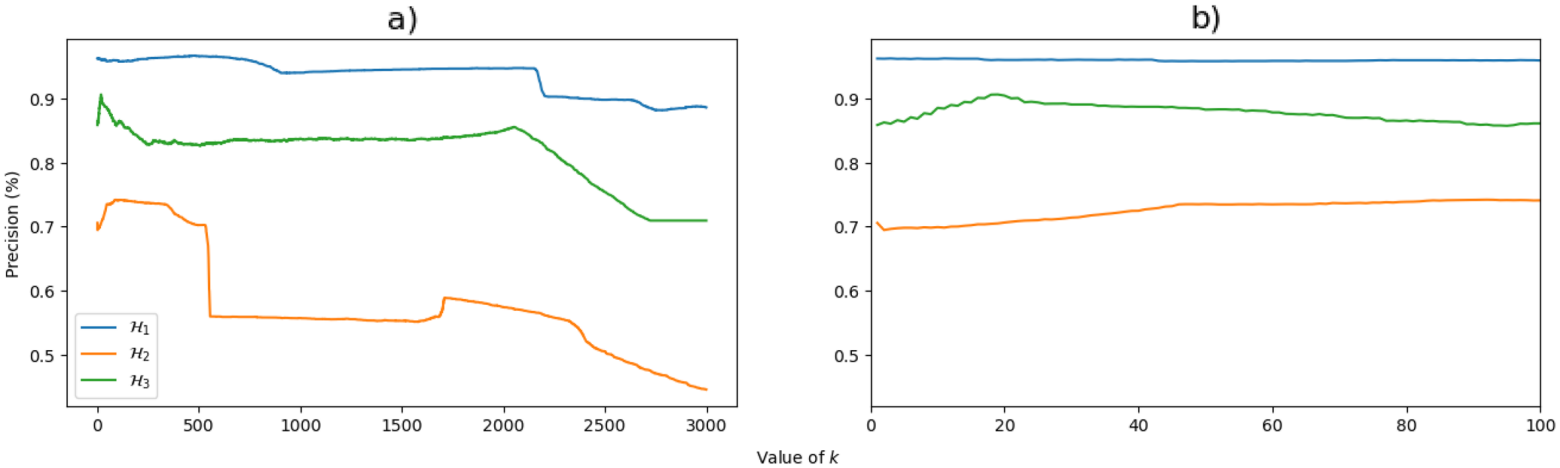
Accuracy obtained as a function of the value of *k* when classifying $\mathcal{H}_1$ using data from $\mathcal{H}_2$ and $\mathcal{H}_3$ for training, when classifying $\mathcal{H}_2$ using data from $\mathcal{H}_1$ and $\mathcal{H}_3$ for training, and when classifying $\mathcal{H}_3$ using data from $\mathcal{H}_1$ and $\mathcal{H}_2$ for training. a) The precision variation for *k* up to $k=3000$, while b) is an inset in the region between $0<k<100$.

Moreover, despite unraveling phase transitions is an subject of great interest in the literature, the method used here is not focused on revealing information about this aspect. The closest to the transitions that this work touches is the idea of identifying their boundaries, given a very high precision in the classification, such as in $$\mathcal{H}_1$$. Lastly, another important discussion is the relationship between the *k* value chosen for the algorithm (the only parameter specified) and the accuracy obtained. This issue is addressed in Fig. 3, which is a graphical representation of the relationship between the accuracy obtained by the algorithm and the number *k* of neighbors used. From this, our analysis show that in our case, the algorithm works better for values between $$30<k<60$$, since for very small values, it becomes more susceptible to noise and, in the case of large values of *k*, distinct phase boundaries are included and the classification of the phases becomes more difficult. In the inset of Fig. 3-b, we can observe the region where $$0<k<100$$, where we see that the precision in each individual model varies according to the value of *k*, so that the region around $$ k=50$$ was chosen because this is where the sum of accuracy when predicting the three combinations is the greatest.

## Conclusion

We have developed a method for studying the phases of unknown magnetic systems through spin correlations. We show that raw correlation data carry information about the phases, which is independent of the model. With the spin correlation information, we use a *k*-NN algorithm to predict the phases of an unknown model with high accuracy. We present a proof of concept, showing that an ML algorithm can classify unknown phases of a Hamiltonian through known phases of another Hamiltonian, creating a model-independent quantum phase classifier. Our strategy tackles the problem with tools that are reproducible and generalizable to new Hamiltonians and, although it does not draw a sharp line to determine the transition points, it provides a faithful outline of the quantum phase diagram for an arbitrary model. A possible advantage of this method is that one can train from a set of numerically less demanding models, and the evaluation can be done on more challenging ones. We emphasize that no explicit use of the phase order parameters is made, so this model-independent classifier opens up the possibility of creating a universal classifier, as more and more model-independent information is added to the classifier database. For future research, we mention the use of explainable artificial intelligence, in an attempt to explain the key features of each phase and provide deeper physical insight.

## Data Availability

The datasets generated during and/or analysed during the current study are available from the corresponding author on reasonable request.
